# Tetra-μ_2_-acetato-bis­{μ_2_-5-meth­oxy-2-[(2-morpholinoeth­yl)iminio­meth­yl]phenolato}tricadmium(II)

**DOI:** 10.1107/S1600536809029171

**Published:** 2009-07-31

**Authors:** Nooraziah Mohd Lair, Hapipah Mohd Ali, Seik Weng Ng

**Affiliations:** a Department of Chemistry, University of Malaya, 50603 Kuala Lumpur, Malaysia

## Abstract

The central Cd^II^ atom in the trinuclear title compound, [Cd_3_(C_14_H_19_N_2_O_3_)_2_(CH_3_COO)_4_], lies on a center of inversion and is bonded to the O atoms of four acetate groups as well as to the phenolate O atoms of the mono-deprotonated Schiff base ligands in a distorted all-*trans* octa­hedral geometry. Two of the acetate groups function in a μ_2_-bridging mode, while the other two each chelate to the terminal Cd^II^ atom and simultaneously bind to the central metal atom in a κ_3_-bonding mode. The Schiff base anions *N*,*O*-chelate to the terminal metal atoms. The morpholine ring assumes a chair conformation.

## Related literature

The Schiff base exists in the zwitterionic form; see: Mohd Lair *et al.* (2009 ▶).
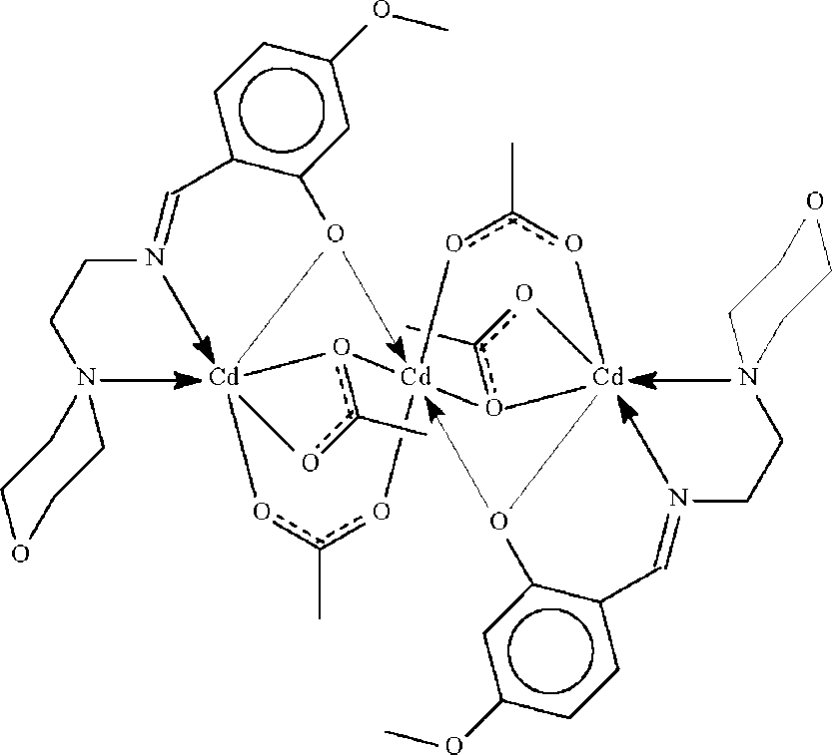


## Experimental

### 

#### Crystal data

[Cd_3_(C_14_H_19_N_2_O_3_)_2_(C_2_H_3_O_2_)_4_]
*M* *_r_* = 1100.00
Triclinic, 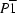

*a* = 8.7199 (1) Å
*b* = 10.5536 (1) Å
*c* = 11.5202 (2) Å
α = 84.899 (1)°
β = 86.317 (1)°
γ = 85.121 (1)°
*V* = 1050.42 (2) Å^3^
*Z* = 1
Mo *K*α radiation
μ = 1.57 mm^−1^
*T* = 193 K
0.30 × 0.25 × 0.20 mm

#### Data collection

Bruker SMART APEX diffractometer
Absorption correction: multi-scan (*SADABS*; Sheldrick, 1996 ▶) *T*_min_ = 0.662, *T*_max_ = 0.730
7364 measured reflections
4655 independent reflections
4265 reflections with *I* > 2σ(*I*)
*R*_int_ = 0.013

#### Refinement

*R*[*F* ^2^ > 2σ(*F* ^2^)] = 0.021
*wR*(*F* ^2^) = 0.071
*S* = 1.12
4655 reflections
262 parameters
H-atom parameters constrained
Δρ_max_ = 0.77 e Å^−3^
Δρ_min_ = −0.45 e Å^−3^

### 

Data collection: *APEX2* (Bruker, 2008 ▶); cell refinement: *SAINT* (Bruker, 2008 ▶); data reduction: *SAINT*; program(s) used to solve structure: *SHELXS97* (Sheldrick, 2008 ▶); program(s) used to refine structure: *SHELXL97* (Sheldrick, 2008 ▶); molecular graphics: *X-SEED* (Barbour, 2001 ▶); software used to prepare material for publication: *publCIF* (Westrip, 2009 ▶).

## Supplementary Material

Crystal structure: contains datablocks global, I. DOI: 10.1107/S1600536809029171/xu2565sup1.cif

Structure factors: contains datablocks I. DOI: 10.1107/S1600536809029171/xu2565Isup2.hkl

Additional supplementary materials:  crystallographic information; 3D view; checkCIF report

## Figures and Tables

**Fig. 1.**
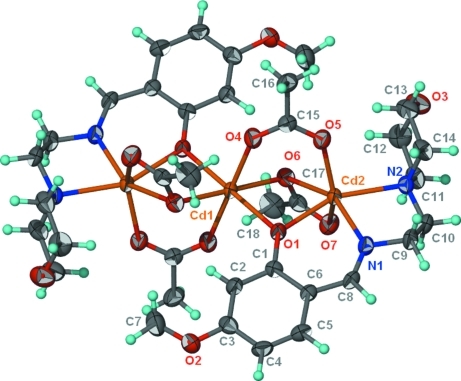
Thermal ellipsoid plot (Barbour, 2001) of Cd3(C2H3O2)4(C13H19N2O3)2 at the 70% probability level. Hydrogen atoms are drawn as spheres of arbitrary radius.

## supplementary crystallographic information

### Experimental

The Schiff base was synthesized as described (Mohd Lair *et al.*, 2009). The Schiff base (0.52 g, 2 mmol) and cadmium(II) acetate dihydrate (0.27 g, 1 mmol) were heated in ethanol (50 ml) for 5 hours. Large crystals appeared after a day.

### Refinement

Hydrogen atoms were placed at calculated positions (C–H 0.95–0.98 Å) and were treated as riding on their parent carbon atoms, with *U*(H) set to 1.2–1.5 times *U*_eq_(*C*).

### Crystal data

**Table:**

[Cd_3_(C_14_H_19_N_2_O_3_)_2_(C_2_H_3_O_2_)_4_]	*Z* = 1
*M**_r_* = 1100.00	*F*(000) = 550
Triclinic, *P*1	*D*_x_ = 1.739 Mg m^−^^3^
Hall symbol: -P 1	Mo *K*α radiation, λ = 0.71073 Å
*a* = 8.7199 (1) Å	Cell parameters from 5715 reflections
*b* = 10.5536 (1) Å	θ = 2.5–28.3°
*c* = 11.5202 (2) Å	µ = 1.57 mm^−^^1^
α = 84.899 (1)°	*T* = 193 K
β = 86.317 (1)°	Prism, colorless
γ = 85.121 (1)°	0.30 × 0.25 × 0.20 mm
*V* = 1050.42 (2) Å^3^	

### Data collection

**Table:**

Bruker SMART APEX diffractometer	4655 independent reflections
Radiation source: fine-focus sealed tube	4265 reflections with *I* > 2σ(*I*)
graphite	*R*_int_ = 0.013
ω scans	θ_max_ = 27.5°, θ_min_ = 1.8°
Absorption correction: multi-scan (*SADABS*; Sheldrick, 1996)	*h* = −11→11
*T*_min_ = 0.662, *T*_max_ = 0.730	*k* = −13→13
7364 measured reflections	*l* = −14→14

### Refinement

**Table:**

Refinement on *F*^2^	Primary atom site location: structure-invariant direct methods
Least-squares matrix: full	Secondary atom site location: difference Fourier map
*R*[*F*^2^ > 2σ(*F*^2^)] = 0.021	Hydrogen site location: inferred from neighbouring sites
*wR*(*F*^2^) = 0.071	H-atom parameters constrained
*S* = 1.12	*w* = 1/[σ^2^(*F*_o_^2^) + (0.0358*P*)^2^ + 0.8302*P*] where *P* = (*F*_o_^2^ + 2*F*_c_^2^)/3
4655 reflections	(Δ/σ)_max_ = 0.001
262 parameters	Δρ_max_ = 0.77 e Å^−^^3^
0 restraints	Δρ_min_ = −0.45 e Å^−^^3^

### Special details

**Table:**

Geometry. All esds (except the esd in the dihedral angle between two l.s. planes) are estimated using the full covariance matrix. The cell esds are taken into account individually in the estimation of esds in distances, angles and torsion angles; correlations between esds in cell parameters are only used when they are defined by crystal symmetry. An approximate (isotropic) treatment of cell esds is used for estimating esds involving l.s. planes.

### Fractional atomic coordinates and isotropic or equivalent isotropic displacement parameters (Å^2^)

**Table:**

	*x*	*y*	*z*	*U*_iso_*/*U*_eq_	
Cd1	0.5000	0.5000	0.5000	0.02237 (7)	
Cd2	0.43304 (2)	0.760182 (16)	0.651747 (15)	0.02350 (7)	
O1	0.3867 (2)	0.70119 (17)	0.47197 (16)	0.0296 (4)	
O2	0.1319 (2)	0.6600 (2)	0.12402 (17)	0.0355 (4)	
O3	0.6154 (3)	0.7935 (2)	0.99064 (18)	0.0443 (5)	
O4	0.7254 (2)	0.58205 (18)	0.51372 (17)	0.0333 (4)	
O5	0.6883 (2)	0.74953 (18)	0.62123 (17)	0.0317 (4)	
O6	0.4399 (2)	0.54112 (17)	0.69398 (15)	0.0280 (4)	
O7	0.2248 (2)	0.6405 (2)	0.7604 (2)	0.0415 (5)	
N1	0.3167 (3)	0.9434 (2)	0.57277 (19)	0.0272 (4)	
N2	0.4101 (2)	0.8934 (2)	0.81198 (18)	0.0260 (4)	
C1	0.2914 (3)	0.7490 (2)	0.3934 (2)	0.0237 (5)	
C2	0.2618 (3)	0.6773 (2)	0.3010 (2)	0.0264 (5)	
H2	0.3112	0.5939	0.2974	0.032*	
C3	0.1638 (3)	0.7243 (3)	0.2161 (2)	0.0286 (5)	
C4	0.0898 (3)	0.8479 (3)	0.2184 (2)	0.0323 (6)	
H4	0.0229	0.8814	0.1592	0.039*	
C5	0.1164 (3)	0.9187 (3)	0.3075 (2)	0.0308 (5)	
H5	0.0655	1.0018	0.3095	0.037*	
C6	0.2162 (3)	0.8742 (2)	0.3969 (2)	0.0230 (5)	
C7	0.1865 (4)	0.5285 (3)	0.1245 (3)	0.0480 (8)	
H7A	0.1564	0.4940	0.0538	0.072*	
H7B	0.1415	0.4809	0.1935	0.072*	
H7C	0.2991	0.5203	0.1265	0.072*	
C8	0.2340 (3)	0.9619 (2)	0.4834 (2)	0.0256 (5)	
H8	0.1779	1.0430	0.4732	0.031*	
C9	0.3179 (3)	1.0469 (2)	0.6485 (2)	0.0316 (6)	
H9A	0.2357	1.1143	0.6281	0.038*	
H9B	0.4183	1.0849	0.6378	0.038*	
C10	0.2915 (3)	0.9945 (3)	0.7749 (2)	0.0327 (6)	
H10A	0.2906	1.0652	0.8260	0.039*	
H10B	0.1891	0.9598	0.7851	0.039*	
C11	0.3554 (3)	0.8297 (3)	0.9251 (2)	0.0344 (6)	
H11A	0.2624	0.7855	0.9140	0.041*	
H11B	0.3266	0.8947	0.9812	0.041*	
C12	0.4778 (4)	0.7347 (3)	0.9742 (3)	0.0425 (7)	
H12A	0.4385	0.6945	1.0500	0.051*	
H12B	0.5017	0.6667	0.9204	0.051*	
C13	0.6732 (3)	0.8499 (3)	0.8810 (3)	0.0397 (7)	
H13A	0.6990	0.7825	0.8268	0.048*	
H13B	0.7689	0.8904	0.8922	0.048*	
C14	0.5570 (3)	0.9488 (3)	0.8280 (2)	0.0341 (6)	
H14A	0.5372	1.0195	0.8793	0.041*	
H14B	0.5997	0.9844	0.7514	0.041*	
C15	0.7716 (3)	0.6686 (2)	0.5665 (2)	0.0255 (5)	
C16	0.9437 (3)	0.6773 (3)	0.5634 (3)	0.0379 (6)	
H16A	0.9691	0.7247	0.6280	0.057*	
H16B	0.9780	0.7217	0.4891	0.057*	
H16C	0.9957	0.5912	0.5709	0.057*	
C17	0.3027 (3)	0.5403 (3)	0.7425 (2)	0.0278 (5)	
C18	0.2436 (4)	0.4112 (3)	0.7764 (3)	0.0462 (8)	
H18A	0.1515	0.4207	0.8292	0.069*	
H18B	0.3234	0.3554	0.8158	0.069*	
H18C	0.2174	0.3736	0.7062	0.069*	

### Atomic displacement parameters (Å^2^)

**Table:**

	*U*^11^	*U*^22^	*U*^33^	*U*^12^	*U*^13^	*U*^23^
Cd1	0.02474 (13)	0.01789 (12)	0.02484 (13)	0.00088 (9)	−0.00239 (9)	−0.00558 (9)
Cd2	0.02618 (10)	0.02014 (10)	0.02439 (10)	0.00142 (7)	−0.00232 (7)	−0.00558 (7)
O1	0.0379 (10)	0.0225 (9)	0.0286 (9)	0.0071 (7)	−0.0111 (8)	−0.0055 (7)
O2	0.0380 (11)	0.0412 (11)	0.0282 (10)	0.0010 (9)	−0.0065 (8)	−0.0092 (8)
O3	0.0429 (12)	0.0609 (14)	0.0291 (10)	−0.0035 (10)	−0.0071 (9)	−0.0012 (10)
O4	0.0296 (9)	0.0329 (10)	0.0391 (11)	−0.0055 (8)	0.0006 (8)	−0.0118 (8)
O5	0.0266 (9)	0.0311 (10)	0.0383 (10)	−0.0019 (7)	0.0023 (8)	−0.0104 (8)
O6	0.0286 (9)	0.0309 (10)	0.0251 (9)	−0.0055 (7)	0.0008 (7)	−0.0034 (7)
O7	0.0332 (10)	0.0406 (12)	0.0514 (13)	0.0022 (9)	0.0009 (9)	−0.0149 (10)
N1	0.0315 (11)	0.0205 (10)	0.0295 (11)	−0.0001 (8)	−0.0015 (9)	−0.0046 (8)
N2	0.0262 (10)	0.0268 (11)	0.0257 (10)	−0.0025 (8)	0.0008 (8)	−0.0070 (8)
C1	0.0225 (11)	0.0262 (12)	0.0222 (11)	−0.0035 (9)	0.0000 (9)	−0.0002 (9)
C2	0.0271 (12)	0.0245 (12)	0.0273 (12)	0.0026 (9)	−0.0013 (10)	−0.0041 (10)
C3	0.0261 (12)	0.0373 (14)	0.0231 (12)	−0.0061 (10)	−0.0008 (9)	−0.0034 (10)
C4	0.0277 (13)	0.0382 (15)	0.0292 (13)	0.0071 (11)	−0.0064 (10)	0.0013 (11)
C5	0.0263 (12)	0.0305 (14)	0.0337 (14)	0.0030 (10)	−0.0021 (10)	0.0021 (11)
C6	0.0222 (11)	0.0219 (11)	0.0242 (11)	−0.0008 (9)	0.0012 (9)	−0.0004 (9)
C7	0.059 (2)	0.0459 (19)	0.0424 (18)	−0.0046 (15)	−0.0072 (15)	−0.0164 (15)
C8	0.0251 (11)	0.0188 (11)	0.0314 (13)	0.0018 (9)	0.0018 (10)	−0.0007 (9)
C9	0.0381 (14)	0.0213 (12)	0.0359 (14)	0.0021 (10)	−0.0030 (11)	−0.0087 (11)
C10	0.0342 (14)	0.0279 (13)	0.0361 (14)	0.0061 (11)	−0.0005 (11)	−0.0120 (11)
C11	0.0366 (14)	0.0442 (16)	0.0238 (12)	−0.0106 (12)	0.0060 (10)	−0.0080 (11)
C12	0.0487 (18)	0.0447 (17)	0.0333 (15)	−0.0058 (14)	0.0017 (13)	0.0009 (13)
C13	0.0323 (14)	0.0550 (19)	0.0324 (14)	−0.0041 (13)	−0.0029 (11)	−0.0055 (13)
C14	0.0349 (14)	0.0388 (15)	0.0305 (13)	−0.0115 (12)	−0.0021 (11)	−0.0063 (11)
C15	0.0241 (11)	0.0246 (12)	0.0273 (12)	−0.0030 (9)	−0.0001 (9)	0.0011 (10)
C16	0.0244 (13)	0.0329 (15)	0.0569 (19)	−0.0022 (11)	0.0007 (12)	−0.0078 (13)
C17	0.0291 (12)	0.0326 (14)	0.0226 (12)	−0.0047 (10)	−0.0038 (9)	−0.0038 (10)
C18	0.0522 (19)	0.0444 (18)	0.0429 (17)	−0.0204 (15)	0.0008 (14)	0.0043 (14)

### Geometric parameters (Å, °)

**Table:**

Cd1—O4	2.2341 (18)	C4—C5	1.364 (4)
Cd1—O4^i^	2.2341 (18)	C4—H4	0.9500
Cd1—O1	2.2697 (18)	C5—C6	1.414 (3)
Cd1—O1^i^	2.2697 (18)	C5—H5	0.9500
Cd1—O6^i^	2.3324 (18)	C6—C8	1.443 (3)
Cd1—O6	2.3324 (18)	C7—H7A	0.9800
Cd2—O5	2.2258 (18)	C7—H7B	0.9800
Cd2—N1	2.251 (2)	C7—H7C	0.9800
Cd2—O1	2.2848 (18)	C8—H8	0.9500
Cd2—O6	2.3163 (18)	C9—C10	1.521 (4)
Cd2—N2	2.406 (2)	C9—H9A	0.9900
Cd2—O7	2.505 (2)	C9—H9B	0.9900
Cd2—C17	2.763 (3)	C10—H10A	0.9900
O1—C1	1.307 (3)	C10—H10B	0.9900
O2—C3	1.364 (3)	C11—C12	1.503 (4)
O2—C7	1.428 (4)	C11—H11A	0.9900
O3—C13	1.428 (4)	C11—H11B	0.9900
O3—C12	1.427 (4)	C12—H12A	0.9900
O4—C15	1.249 (3)	C12—H12B	0.9900
O5—C15	1.259 (3)	C13—C14	1.508 (4)
O6—C17	1.288 (3)	C13—H13A	0.9900
O7—C17	1.232 (3)	C13—H13B	0.9900
N1—C8	1.286 (3)	C14—H14A	0.9900
N1—C9	1.458 (3)	C14—H14B	0.9900
N2—C10	1.476 (3)	C15—C16	1.508 (3)
N2—C14	1.481 (3)	C16—H16A	0.9800
N2—C11	1.483 (3)	C16—H16B	0.9800
C1—C2	1.409 (3)	C16—H16C	0.9800
C1—C6	1.427 (4)	C17—C18	1.508 (4)
C2—C3	1.372 (4)	C18—H18A	0.9800
C2—H2	0.9500	C18—H18B	0.9800
C3—C4	1.407 (4)	C18—H18C	0.9800
			
O4—Cd1—O4^i^	180.00 (9)	C6—C5—H5	118.4
O4—Cd1—O1	89.08 (7)	C5—C6—C1	118.0 (2)
O4^i^—Cd1—O1	90.92 (7)	C5—C6—C8	116.2 (2)
O4—Cd1—O1^i^	90.92 (7)	C1—C6—C8	125.9 (2)
O4^i^—Cd1—O1^i^	89.08 (7)	O2—C7—H7A	109.5
O1—Cd1—O1^i^	180.0	O2—C7—H7B	109.5
O4—Cd1—O6^i^	92.07 (7)	H7A—C7—H7B	109.5
O4^i^—Cd1—O6^i^	87.93 (7)	O2—C7—H7C	109.5
O1—Cd1—O6^i^	99.40 (7)	H7A—C7—H7C	109.5
O1^i^—Cd1—O6^i^	80.60 (7)	H7B—C7—H7C	109.5
O4—Cd1—O6	87.93 (7)	N1—C8—C6	127.7 (2)
O4^i^—Cd1—O6	92.07 (7)	N1—C8—H8	116.2
O1—Cd1—O6	80.60 (7)	C6—C8—H8	116.2
O1^i^—Cd1—O6	99.40 (7)	N1—C9—C10	109.2 (2)
O6^i^—Cd1—O6	180.000 (1)	N1—C9—H9A	109.8
O5—Cd2—N1	112.77 (8)	C10—C9—H9A	109.8
O5—Cd2—O1	95.22 (7)	N1—C9—H9B	109.8
N1—Cd2—O1	79.57 (7)	C10—C9—H9B	109.8
O5—Cd2—O6	90.89 (7)	H9A—C9—H9B	108.3
N1—Cd2—O6	150.28 (7)	N2—C10—C9	113.0 (2)
O1—Cd2—O6	80.63 (6)	N2—C10—H10A	109.0
O5—Cd2—N2	98.35 (7)	C9—C10—H10A	109.0
N1—Cd2—N2	77.40 (8)	N2—C10—H10B	109.0
O1—Cd2—N2	156.39 (7)	C9—C10—H10B	109.0
O6—Cd2—N2	118.22 (7)	H10A—C10—H10B	107.8
O5—Cd2—O7	140.68 (7)	N2—C11—C12	111.1 (2)
N1—Cd2—O7	106.34 (8)	N2—C11—H11A	109.4
O1—Cd2—O7	95.62 (7)	C12—C11—H11A	109.4
O6—Cd2—O7	54.04 (7)	N2—C11—H11B	109.4
N2—Cd2—O7	86.00 (7)	C12—C11—H11B	109.4
O5—Cd2—C17	117.38 (8)	H11A—C11—H11B	108.0
N1—Cd2—C17	128.89 (8)	O3—C12—C11	111.6 (3)
O1—Cd2—C17	86.63 (7)	O3—C12—H12A	109.3
O6—Cd2—C17	27.64 (7)	C11—C12—H12A	109.3
N2—Cd2—C17	103.91 (7)	O3—C12—H12B	109.3
O7—Cd2—C17	26.48 (7)	C11—C12—H12B	109.3
C1—O1—Cd1	129.37 (16)	H12A—C12—H12B	108.0
C1—O1—Cd2	132.41 (16)	O3—C13—C14	111.5 (2)
Cd1—O1—Cd2	95.61 (7)	O3—C13—H13A	109.3
C3—O2—C7	117.8 (2)	C14—C13—H13A	109.3
C13—O3—C12	109.2 (2)	O3—C13—H13B	109.3
C15—O4—Cd1	135.87 (17)	C14—C13—H13B	109.3
C15—O5—Cd2	127.15 (16)	H13A—C13—H13B	108.0
C17—O6—Cd2	95.85 (16)	N2—C14—C13	111.4 (2)
C17—O6—Cd1	123.51 (15)	N2—C14—H14A	109.4
Cd2—O6—Cd1	93.08 (6)	C13—C14—H14A	109.4
C17—O7—Cd2	88.50 (16)	N2—C14—H14B	109.4
C8—N1—C9	118.1 (2)	C13—C14—H14B	109.4
C8—N1—Cd2	129.16 (18)	H14A—C14—H14B	108.0
C9—N1—Cd2	111.79 (16)	O4—C15—O5	126.2 (2)
C10—N2—C14	110.6 (2)	O4—C15—C16	116.7 (2)
C10—N2—C11	108.4 (2)	O5—C15—C16	117.1 (2)
C14—N2—C11	108.7 (2)	C15—C16—H16A	109.5
C10—N2—Cd2	102.71 (15)	C15—C16—H16B	109.5
C14—N2—Cd2	111.70 (16)	H16A—C16—H16B	109.5
C11—N2—Cd2	114.69 (16)	C15—C16—H16C	109.5
O1—C1—C2	120.1 (2)	H16A—C16—H16C	109.5
O1—C1—C6	121.9 (2)	H16B—C16—H16C	109.5
C2—C1—C6	118.0 (2)	O7—C17—O6	121.3 (2)
C3—C2—C1	122.0 (2)	O7—C17—C18	122.2 (3)
C3—C2—H2	119.0	O6—C17—C18	116.6 (3)
C1—C2—H2	119.0	O7—C17—Cd2	65.02 (15)
O2—C3—C2	124.6 (3)	O6—C17—Cd2	56.51 (13)
O2—C3—C4	114.9 (2)	C18—C17—Cd2	171.3 (2)
C2—C3—C4	120.4 (2)	C17—C18—H18A	109.5
C5—C4—C3	118.5 (2)	C17—C18—H18B	109.5
C5—C4—H4	120.8	H18A—C18—H18B	109.5
C3—C4—H4	120.8	C17—C18—H18C	109.5
C4—C5—C6	123.1 (3)	H18A—C18—H18C	109.5
C4—C5—H5	118.4	H18B—C18—H18C	109.5
			
O4—Cd1—O1—C1	132.7 (2)	O5—Cd2—N2—C14	11.13 (18)
O4^i^—Cd1—O1—C1	−47.3 (2)	N1—Cd2—N2—C14	−100.46 (18)
O6^i^—Cd1—O1—C1	40.8 (2)	O1—Cd2—N2—C14	−113.3 (2)
O6—Cd1—O1—C1	−139.2 (2)	O6—Cd2—N2—C14	106.68 (17)
O4—Cd1—O1—Cd2	−64.07 (7)	O7—Cd2—N2—C14	151.81 (18)
O4^i^—Cd1—O1—Cd2	115.93 (7)	C17—Cd2—N2—C14	132.15 (17)
O6^i^—Cd1—O1—Cd2	−156.02 (7)	O5—Cd2—N2—C11	−113.06 (18)
O6—Cd1—O1—Cd2	23.98 (7)	N1—Cd2—N2—C11	135.35 (19)
O5—Cd2—O1—C1	−131.7 (2)	O1—Cd2—N2—C11	122.5 (2)
N1—Cd2—O1—C1	−19.5 (2)	O6—Cd2—N2—C11	−17.5 (2)
O6—Cd2—O1—C1	138.2 (2)	O7—Cd2—N2—C11	27.61 (18)
N2—Cd2—O1—C1	−6.7 (3)	C17—Cd2—N2—C11	7.95 (19)
O7—Cd2—O1—C1	86.1 (2)	Cd1—O1—C1—C2	−8.6 (3)
C17—Cd2—O1—C1	111.1 (2)	Cd2—O1—C1—C2	−165.66 (17)
O5—Cd2—O1—Cd1	65.89 (8)	Cd1—O1—C1—C6	172.28 (16)
N1—Cd2—O1—Cd1	178.12 (8)	Cd2—O1—C1—C6	15.2 (3)
O6—Cd2—O1—Cd1	−24.16 (7)	O1—C1—C2—C3	−179.3 (2)
N2—Cd2—O1—Cd1	−169.14 (14)	C6—C1—C2—C3	−0.2 (4)
O7—Cd2—O1—Cd1	−76.26 (8)	C7—O2—C3—C2	9.3 (4)
C17—Cd2—O1—Cd1	−51.32 (8)	C7—O2—C3—C4	−171.9 (3)
O1—Cd1—O4—C15	46.8 (3)	C1—C2—C3—O2	179.3 (2)
O1^i^—Cd1—O4—C15	−133.2 (3)	C1—C2—C3—C4	0.5 (4)
O6^i^—Cd1—O4—C15	146.2 (3)	O2—C3—C4—C5	−179.7 (2)
O6—Cd1—O4—C15	−33.8 (3)	C2—C3—C4—C5	−0.8 (4)
N1—Cd2—O5—C15	−118.7 (2)	C3—C4—C5—C6	0.7 (4)
O1—Cd2—O5—C15	−37.8 (2)	C4—C5—C6—C1	−0.4 (4)
O6—Cd2—O5—C15	42.9 (2)	C4—C5—C6—C8	178.8 (2)
N2—Cd2—O5—C15	161.6 (2)	O1—C1—C6—C5	179.2 (2)
O7—Cd2—O5—C15	67.7 (2)	C2—C1—C6—C5	0.1 (3)
C17—Cd2—O5—C15	51.1 (2)	O1—C1—C6—C8	0.1 (4)
O5—Cd2—O6—C17	164.09 (15)	C2—C1—C6—C8	−179.0 (2)
N1—Cd2—O6—C17	−52.0 (2)	C9—N1—C8—C6	179.6 (2)
O1—Cd2—O6—C17	−100.76 (14)	Cd2—N1—C8—C6	−12.3 (4)
N2—Cd2—O6—C17	64.12 (16)	C5—C6—C8—N1	179.5 (2)
O7—Cd2—O6—C17	3.27 (14)	C1—C6—C8—N1	−1.4 (4)
O5—Cd2—O6—Cd1	−71.76 (7)	C8—N1—C9—C10	131.7 (2)
N1—Cd2—O6—Cd1	72.13 (15)	Cd2—N1—C9—C10	−38.4 (3)
O1—Cd2—O6—Cd1	23.38 (7)	C14—N2—C10—C9	73.7 (3)
N2—Cd2—O6—Cd1	−171.73 (6)	C11—N2—C10—C9	−167.4 (2)
O7—Cd2—O6—Cd1	127.41 (9)	Cd2—N2—C10—C9	−45.6 (2)
C17—Cd2—O6—Cd1	124.14 (16)	N1—C9—C10—N2	59.6 (3)
O4—Cd1—O6—C17	165.0 (2)	C10—N2—C11—C12	−174.1 (2)
O4^i^—Cd1—O6—C17	−15.0 (2)	C14—N2—C11—C12	−53.9 (3)
O1—Cd1—O6—C17	75.5 (2)	Cd2—N2—C11—C12	71.9 (3)
O1^i^—Cd1—O6—C17	−104.5 (2)	C13—O3—C12—C11	−59.8 (3)
O4—Cd1—O6—Cd2	65.86 (7)	N2—C11—C12—O3	58.4 (3)
O4^i^—Cd1—O6—Cd2	−114.14 (7)	C12—O3—C13—C14	59.3 (3)
O1—Cd1—O6—Cd2	−23.55 (7)	C10—N2—C14—C13	172.4 (2)
O1^i^—Cd1—O6—Cd2	156.45 (7)	C11—N2—C14—C13	53.7 (3)
O5—Cd2—O7—C17	−34.6 (2)	Cd2—N2—C14—C13	−73.9 (2)
N1—Cd2—O7—C17	151.47 (16)	O3—C13—C14—N2	−57.7 (3)
O1—Cd2—O7—C17	70.73 (16)	Cd1—O4—C15—O5	−9.5 (4)
O6—Cd2—O7—C17	−3.40 (14)	Cd1—O4—C15—C16	170.9 (2)
N2—Cd2—O7—C17	−132.91 (17)	Cd2—O5—C15—O4	3.8 (4)
O5—Cd2—N1—C8	108.5 (2)	Cd2—O5—C15—C16	−176.71 (18)
O1—Cd2—N1—C8	17.2 (2)	Cd2—O7—C17—O6	5.8 (2)
O6—Cd2—N1—C8	−31.8 (3)	Cd2—O7—C17—C18	−174.5 (2)
N2—Cd2—N1—C8	−157.7 (2)	Cd2—O6—C17—O7	−6.3 (3)
O7—Cd2—N1—C8	−75.7 (2)	Cd1—O6—C17—O7	−103.9 (3)
C17—Cd2—N1—C8	−59.8 (3)	Cd2—O6—C17—C18	174.0 (2)
O5—Cd2—N1—C9	−82.79 (18)	Cd1—O6—C17—C18	76.3 (3)
O1—Cd2—N1—C9	−174.11 (18)	Cd1—O6—C17—Cd2	−97.62 (15)
O6—Cd2—N1—C9	136.93 (17)	O5—Cd2—C17—O7	156.09 (15)
N2—Cd2—N1—C9	11.08 (17)	N1—Cd2—C17—O7	−36.1 (2)
O7—Cd2—N1—C9	93.04 (18)	O1—Cd2—C17—O7	−109.77 (16)
C17—Cd2—N1—C9	108.92 (18)	O6—Cd2—C17—O7	174.1 (2)
O5—Cd2—N2—C10	129.62 (16)	N2—Cd2—C17—O7	48.83 (17)
N1—Cd2—N2—C10	18.03 (15)	O5—Cd2—C17—O6	−17.97 (16)
O1—Cd2—N2—C10	5.2 (3)	N1—Cd2—C17—O6	149.87 (13)
O6—Cd2—N2—C10	−134.83 (15)	O1—Cd2—C17—O6	76.16 (14)
O7—Cd2—N2—C10	−89.70 (16)	N2—Cd2—C17—O6	−125.24 (14)
C17—Cd2—N2—C10	−109.37 (16)	O7—Cd2—C17—O6	−174.1 (2)

Symmetry codes: (i) −*x*+1, −*y*+1, −*z*+1.
